# A nucleobase-binding pocket in a viral RNA-dependent RNA polymerase contributes to elongation complex stability

**DOI:** 10.1093/nar/gkz1170

**Published:** 2019-12-21

**Authors:** Wei Shi, Han-Qing Ye, Cheng-Lin Deng, Rui Li, Bo Zhang, Peng Gong

**Affiliations:** 1 Key Laboratory of Special Pathogens and Biosafety, Wuhan Institute of Virology, Center for Biosafety Mega-Science, Chinese Academy of Sciences, No.44 Xiao Hong Shan, Wuhan, Hubei 430071, China; 2 University of Chinese Academy of Sciences, Beijing 100049, China; 3 Drug Discovery Center for Infectious Diseases, Nankai University, Tianjin 300350, China

## Abstract

The enterovirus 71 (EV71) 3D^pol^ is an RNA-dependent RNA polymerase (RdRP) that plays the central role in the viral genome replication, and is an important target in antiviral studies. Here, we report a crystal structure of EV71 3D^pol^ elongation complex (EC) at 1.8 Å resolution. The structure reveals that the 5′-end guanosine of the downstream RNA template interacts with a fingers domain pocket, with the base sandwiched by H44 and R277 side chains through hydrophobic stacking interactions, and these interactions are still maintained after one in-crystal translocation event induced by nucleotide incorporation, implying that the pocket could regulate the functional properties of the polymerase by interacting with RNA. When mutated, residue R277 showed an impact on virus proliferation in virological studies with residue H44 having a synergistic effect. *In vitro* biochemical data further suggest that mutations at these two sites affect RNA binding, EC stability, but not polymerase catalytic rate (*k*_cat_) and apparent NTP affinity (*K*_M,NTP_). We propose that, although rarely captured by crystallography, similar surface pocket interaction with nucleobase may commonly exist in nucleic acid motor enzymes to facilitate their processivity. Potential applications in antiviral drug and vaccine development are also discussed.

## INTRODUCTION

Human enterovirus 71 (EV71), a member of the *Enterovirus A* (EV_A) species belonging to the *Enterovirus* genus of the *Picornaviridae* family, is one of the major causative agents of hand, foot and mouth disease (HFMD), and can sometimes cause severe neurological complications such as encephalitis, aseptic meningitis, and acute flaccid paralysis (1–3). The genome of EV71 is a single-stranded positive-sense RNA of approximately 7400 nucleotides (nt) encoding a 240-kD polyprotein. The polyprotein is co- and/or post-translationally processed by host or viral proteases into four structural proteins (VP1-VP4) and seven non-structural proteins (2A-2C and 3A-3D) (4). The 3D (also known as 3D^pol^) protein is an RNA-dependent RNA polymerase (RdRP) that plays the central role in viral RNA genome replication, and has become a major target for antiviral studies. To date, numerous crystal structures of picornavirus RdRP proteins have been solved. Along with RdRPs from other RNA viruses, the polymerase catalytic core adopts an encircled right hand architecture with palm, fingers and thumb domains surrounding the active site (5–7). The fingers domain can be further divided into index, middle, ring and pinky subdomains following a nomenclature first utilized in describing the poliovirus (PV, a member of the *Enterovirus* C species) RdRP (6). The index finger subdomain interacts with the thumb to make the hallmark encirclement distinguished from other classes of processive polymerases (5–6,8). A collection of viral RdRP structures in complex with RNA or with RNA/NTP further have illustrated the mechanism of the RdRP catalytic mechanisms, in particular, the mechanism of the polymerase nucleotide addition cycle (NAC) with unique features in the pre-catalysis active site closure and post-catalysis translocation events (9–13).

Compared to the relatively conserved catalytic mechanisms, the regulatory mechanisms of viral RdRPs are quite diverse. Other viral proteins, RNA elements, host factors or even regions as part of the RdRP molecule could affect the function of RdRP during the entire genome replication process. The key interactions with the RdRP catalytic core involved in these regulation processes can be classified into two major types: one from other molecules and the other from region(s) that are part of the RdRP molecule but are beyond the RdRP catalytic core. Examples of the first type include the regulation of flavivirus NS5 by viral protein NS3 and viral RNA elements within the 5′-untranslated region (UTR) (14–16), picornavirus 3D^pol^ by viral protein 3CD and host SUMOylation (SUMO stands for small ubiquitin-like modifier) machineries (17,18), and coronavirus nsp12 by nsp7 and nsp8 (19). Representative cases of the second type have been identified in the *Flaviviridae* RdRPs, with the methyltransferase (MTase, part of the flavivirus NS5) and the N-terminal domain (NTD, part of the pestivirus NS5B) regulating the corresponding polymerases, respectively, through intra-molecular interactions (20–25).

In this work, we identified and characterized a type of regulatory interaction that is quite unique if compared with the aforementioned interaction types. These interactions only involve a small surface pocket within the RdRP fingers domain and a nucleobase of the RNA that is part of RdRP–RNA catalytic complex. We unintentionally identified these interactions when solving an EV71 RdRP elongation complex (EC) crystal structure. Through a set of biochemical characterizations, we further demonstrated that this surface pocket facilitates EC stability, likely through its interactions with the nucleobase within the RdRP–RNA complex, while the elongation catalytic rates are not affected. Cell-based virological data comparing the wild type (WT) and mutant viruses further suggest that this pocket is important to virus proliferation. To the best of our knowledge, regulatory sites within the RdRP catalytic core that interact with the downstream RNA have not been reported with support of structural data. Our study provides an example of evolution and utilization of nucleobase anchoring sites by viral RdRPs to facilitate or maintain their essential catalytic properties, and the identified surface pocket may also serve as an intervention target for developing antiviral applications.

## MATERIALS AND METHODS

### Plasmid construction, protein expression and protein purification

The EV71 3D^pol^ gene within the DNA clone of strains HeN09-17/HeN/CHN2009 (GenBank accession no. JX678881, genotype C) and BrCr-ts (GenBank accession no: AB204853.1, genotype A) was cloned into a pET26b-Ub vector (26,27). The resulting plasmids were transformed into *Escherichia coli* strain BL21(DE3)pCG1 (kindly provided by Dr Craig Cameron, Pennsylvania State University, State College, PA, USA) for the production of the full-length 3D^pol^ protein with a native glycine at its N-terminus according to previously described methods and a C-terminal hexa-histidine tag for affinity purification (26,28). All 3D^pol^ point mutations were introduced by using the QuickChange site-directed mutagenesis method and the corresponding WT plasmid as the template (29). Cell growth, isopropyl-β-D-thiogalactopyranoside (IPTG) induction, cell harvesting, cell lysis, protein purification and protein storage were performed as described previously (10,28), except that the temperature for overnight culture growth was 37°C. The final buffer condition for protein storage was 5 mM Tris (pH 7.5), 200 mM NaCl, 0.02% (wt./vol.) NaN_3_, and 5 mM Tris(2-carboxyethyl)phosphine (TCEP). 3D^pol^ concentrations were measured by absorbance at 280 nm using an extinction coefficient of 71 280 M^−1^cm^−1^ calculated by the ExPASy ProtParam program (http://web.expasy.org/protparam/). The typical protein yield was 10–15 mg per liter of bacteria culture.

### RNA preparation and 3D^pol^ EC assembly

The 31-mer template RNA (T31) was prepared by *in vitro* T7 RNA polymerase transcription and subsequent glmS ribozyme cleavage according to protocols described previously (30,31). T31 was subjected to a self-annealing process before being annealed to an 8-mer RNA (P8, Integrated DNA technologies) at a 1:1.1 molar ratio to yield the T31/P8 construct according to protocols described previously (31). The EV71 3D^pol^ EC assembly, purification, and storage were carried out using protocols described previously (10), except that the assembly reaction was conducted in a buffer containing 50 mM HEPES (pH 7.0), 50 mM NaCl, 75 mM KCl, 8 mM MgCl_2_ and 4 mM TCEP.

### EC crystallization, NTP soaking of the EC crystals and crystal harvesting

The EC crystals were grown by sitting-drop vapor diffusion at 16°C using a 12.5 mg/ml EC sample. Rhombohedron-shape crystals grew to their final size within 1–2 weeks in a precipitant solution containing 0.17 M sodium acetate, 0.085 M Tris (pH 8.5), 25.5% (wt./vol.) PEG4000 and 15% (vol./vol.) glycerol. Crystal soaking trials were done for 10 h using the precipitant solution supplemented with 5 mM ddCTP and 10 mM MgCl_2_. Crystals were directly cooled and stored in liquid nitrogen prior to data collection.

### Crystallographic data processing and structure determination

X-ray diffraction data was collected at Shanghai Synchrotron Radiation Facility (SSRF) beamline BL17U1 (wavelength: 0.9792 Å, temperature: 100 K). Data of at least 180° were typically collected in 1° oscillation steps. Reflections were integrated, merged and scaled using D*Trek (32). The initial structure solution was obtained using the molecular replacement program PHASER (33) with coordinates derived from EV-B EC structure (PDB entry: 5F8G, chains A-C) as the search model (10). Manual model building and structure refinement were done using Coot and Phenix, respectively (34,35). The 3500-K composite simulated-annealing (SA) omit 2F_o_-F_c_ electron density maps were generated using CNS (36). Unless otherwise indicated, protein structure superpositioning was done using the maximum likelihood-based structure superpositioning program THESEUS (37).

### Fluorescence polarization (FP)-based RNA binding assays

The binding affinity of the WT EV71 3D^pol^ or its variant to the template/primer RNA was assessed using a fluorescence polarization (FP)-based assay. A T31/P8F (with a 1:1.1 molar ratio) construct was used as the RNA substrate. Except for bearing a 6-FAM (6-carboxyfluorescein) at the 5′-end of P8, it is otherwise identical to the P31/P8 construct used in the EC assembly. For each 3D^pol^ construct, a set of 14 mixtures were prepared, each with 25 nM T31/P8F construct (according to T31 concentration) and concentrations of 3D^pol^ in the range of 3.2 nM to 5.6 μM in a FP buffer containing 50 mM potassium glutamate (pH 7.5), 50 mM arginine, 5 mM dithiolthreitol (DTT), 5 mM MgCl_2_, 30 mM NaCl and 5% (vol./vol.) glycerol. The mixtures were loaded into the wells of a black 384-well plate (OptiPlate 384-F, PerkinElmer). After at least 60-min of incubation at room temperature (r.t.), the fluorescent signals were measured in the 2102 EnVision Multilabel reader (PerkinElmer) using an excitation wavelength of 485 nm (monochromator) and a 535-nm high band pass emission filter. The FP values were calculated based on the fluorescent signals parallel (F_∥_) and perpendicular (F_⊥_) to the excitation light and the equation FP = (F_∥_-F_⊥_)/(F_∥_+F_⊥_). The FP values at different 3D^pol^ concentrations were fitted to the one-site binding model for determination of the dissociation constant (*K*_d_) values following the equation FP = offset+amplitude•[3D^pol^]/([3D^pol^]+*K_d_*). Five replicates were obtained for each 3D^pol^ concentration. The FP values were decreased at high-concentration data points, possibly resulting from protein aggregation and therefore these data points were omitted in fitting routines.

### EC assembly assays

To characterize the process of EC formation for WT EV71 3D^pol^ and its variants, the EC assembly reactions were carried out as described above with a total volume of 20 μl except that the 3D^pol^ and RNA concentrations were 6 and 4 μM, respectively, the KCl concentration was 55 mM, and the NaCl concentration was 20 mM. At each indicated reaction time point, an aliquot of the reaction mixture was withdrawn and mixed with an equal volume of stop solution containing 95% (vol./vol.) formamide, 20 mM EDTA (pH 8.0), and 0.02% (wt./vol.) xylene cyano. The RNA species were analyzed by denaturing polyacrylamide gel electrophoresis (PAGE) followed by Stains-All staining and band intensity quantification as previously described (31). For each 3D^pol^ construct, pre-incubation of 3D^pol^ and T31/P8 at 22.5°C prior to the addition of the NTP mixture for various time were performed in parallel to the experiment without pre-incubation.

### EC stability assays

The EC stability assay were modified from protocols described previously (38). EC assembly reactions were formed as described above for 120 min. NaCl was then added to reach a final concentration of 300 mM to prevent 3D^pol^-RNA rebinding and reinitiation, and the reaction mixture was incubated at 30°C. At various time points after the addition of NaCl, a 10 μl aliquot was withdrawn from the mixture and supplemented with CTP to reach a final CTP concentration of 300 μM. The CTP-triggered reaction proceeded for 3 min at 25°C. Reaction quenching, gel electrophoresis, staining and quantification were performed as described in the EC formation assays (31). Experiments were performed in triplicates for EV71-C 3D^pol^ trials.

### Stopped-flow fluorescence assays for determining 3D^pol^ elongation rate constants

Two types of stopped-flow fluorescence assays were established in measuring the EC elongation rates. The first assay was modified from protocols described previously (39,40). The 11-mer RNA primer (P11L) with a LI-COR label at the 5′-end and the 35-mer RNA template T35F_term_ with a 5′-Fluorescein (Integrated DNA technologies) were annealed at a 1:1 molar ratio to yield the T35F_term_/P11L construct before being used in a stopped-flow assay for measuring the overall 3D^pol^ elongation rates over a stretch of 17 nt. The LI-COR function was not used in this study due to the lack of an appropriate imaging instrument. By modifying the sequence at the 5′ region of the RNA template from previously described methods (39), the fluorescent signal increase was only observed upon the incorporation of the last five nucleotides. Kinetics experiments were performed using a Chriscan SF3 instrument (Applied Photophysics) with equal volume mixing of the pre-assembled EC and elongation NTPs. Fluorescence excitation wavelength was 492 nm (monochromator, bandwidth 2 nm) and fluorescent signal was detected using a 515-nm high band pass filter. ECs were pre-assembled at 22.5°C for 90 min using 1 μM T35F_term_/P11L construct, 8 μM 3D^pol^ and 100 μM each ATP and GTP in a SF buffer containing 50 mM HEPES (pH 7.0), 20 mM NaCl, 55 mM KCl, 5.5 mM MgCl_2_ and 4 mM TCEP. The reaction mixture was then 20-fold diluted using the SF buffer to a reach a final RNA concentration of 50 nM. The fluorescence signal change was measured at 22.5°C after rapid-mixing, producing a final solution with 25 nM RNA, 200 nM 3D^pol^, 150 μM ATP/GTP/CTP and 2.5–160 μM UTP in SF buffer.

Data analysis and fitting were done as described previously (39–41). The data of rapid signal increase observed at the end of the elongation reaction were fitted into a single exponential rise curve. The time of intersection point of this exponential curve with the lag phase signal was taken as a measure of time of elongating 17-nt single-stranded region, not including the five terminal nucleotides that do not contribute to the lag phase. The average elongation rate was calculated for all 17 elongation steps constituting the lag phase. This approximation treatment was demonstrated to reasonably estimate the average elongation rate when compared to more thorough data analysis to model the entire elongation reaction steps as a series of irreversible steps with an equal rate constant (40). The observed elongation rates were then plotted as a function of UTP concentration to determine the *k*_pol_ and apparent *K*_M_ values by fitting the data to the Michaelis–Menten type equation, rate = *k*_pol_•[UTP]/([UTP]+*K*_M_).

The P8 primer and T31-F_int_ template (Integrated DNA technologies) were annealed into duplex mimicking T31/P8 RNA duplex to determine the incorporation rates of a 2′-deoxy-CMP (2dCMP) directed by a templating guanosine 5 nt upstream of the fluorescein (Figure 7D). The stopped-flow routine was performed as described above. The data of rapid signal decrease observed for 2dCMP incorporation were fitted into a single exponential decay curve Y = offset+amplitude•exp(-rate•t). Due to deviation from the single exponential curvature, the latter portion of the signal was omitted in the fitting routine (Figure 7D). The incorporation rates were then plotted as a function of the 2′-deoxy-CTP (2dCTP) concentration, and the data were fitted to the Michaelis–Menten type equation to determine the *k*_pol_ and apparent *K*_M_ values.

### Genome-length EV71 cDNA mutant construction and the immunofluorescence assay (IFA)

EV71 genome-length cDNA clones with different mutations were constructed using the infectious cDNA clone of pACYC-EV71-FL as the backbone. Fusion PCR was used to engineer all mutations into the backbone (27). All constructs were validated by DNA sequencing. RNA transcription, transfection and subsequent IFA were carried out as previously described (27,42)

## RESULTS

### The global structure of the EV71 RdRP EC resembles those of the previously reported enterovirus RdRP ECs except for the placement of the downstream RNA

**Figure 1.**
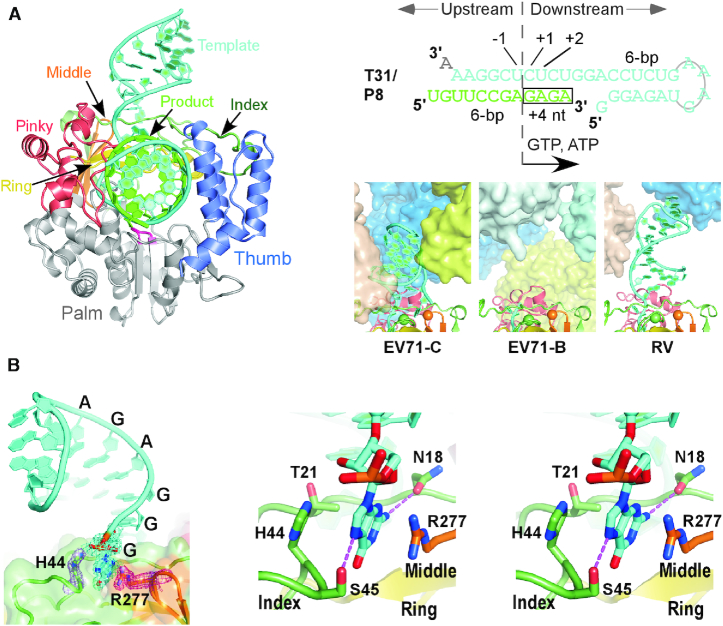
A nucleobase-binding pocket identified in an EV71 RdRP EC crystal structure. (**A**) EC structure (left, viewed into the front channel), RNA construct for EC assembly (top right) and a comparison of downstream RNA in enterovirus RdRP EC structures (bottom right). RNA strands and structural regions of the RdRP are color-coded as indicated. The signature sequence YGDD is in magenta. The boxed region in the RNA sequence indicates the tetra-nucleotide incorporated into the product strand during EC assembly. Neighboring complexes (in different colors) around the downstream RNA of the EC in each crystal lattice were shown in surface representation. The α-carbon of the two key residues participating in the stacking interactions with the nucleobase are shown as spheres. PDB entries used: EV71-C–6KWQ; EV71-B–5F8G; RV (rhinovirus)–4K50. (**B**) The interactions between the pocket and the 5′-nucleobase. The coloring scheme is the same as in panel A. Left: a global view of the interactions with composite SA omit electron density maps (contoured at 1.0 σ) of the terminal guanosine and residues H44 and R277 overlaid. Right: stereo-pair images of the interaction details with key side chains and the 5′-guanosine shown in sticks. Dashed lines indicate hydrogen bonding interactions between the guanine and side chains of residues N18 and S45.

Based on established methods in obtaining picornaviral RdRP EC crystals (43), we assembled a genotype-C EV71 RdRP EC using an RNA construct T31/P8 comprising a 31-nt template (T31) and an 8-nt primer (P8) to direct the incorporation of a GAGA tetra-nucleotide by providing GTP and ATP as the only NTP substrates (Figure 1A), purified the EC by anion-exchange chromatography, and crystallized the EC. The crystal structure was solved at 1.8 Å resolution in space group C2 by molecular replacement using previously reported genotype-B EV71 RdRP EC structure as the search model (Table 1) (10). The final model had *R*_work_ and *R*_free_ values of 0.181 and 0.214, respectively, and the crystallographic asymmetric unit contained only one EC. The RdRP in this EC is structurally consistent with RdRPs in the previously reported enterovirus EC structures (10,43–45) with the root-mean-square deviation (RMSD) values in the range of 0.5–1.1 Å for all superimposable α-carbon atoms (the new structure as the reference; 99–100% coverage). The majority of the RNA is well ordered including all downstream nucleotides that form a stem-loop structure (Figure 1A and B). Based on available structural information, the downstream RNA of the picornaviral RdRP EC is flexible and does not have a consistent conformation (Figure 1A, bottom right) (10,43–45). The crystal contacts could play important roles in stabilizing the RNA in certain conformations and resulting in the high-resolution feature of this EC crystal form. The high-resolution nature of this structure allows identification of a total of five ordered magnesium ions associated with this EC, interacting with the upstream (two ions) and downstream (two ions) RNA and residues of the catalytic motifs A and C in the palm domain (one ion).

**Table 1.** X-ray diffraction data collection and structure refinement statistics

EC form—PDB	Native—6KWQ	ddCTP—6KWR
**Data collection** ^1^		
Space group	C2	C2
Cell dimensions		
*a*, *b*, *c* (Å)	124.3, 75.5, 74.0	130.0, 76.5, 68.1
α, β, γ (°)	90, 91.5, 90	90, 94.9, 90
Resolution (Å)^2^	47.0–1.76 (1.82–1.76)	38.2–2.50 (2.59–2.50)
R_merge_	0.077 (0.43)	0.075 (0.46)
R_meas_	0.090 (0.50)	0.092 (0.57)
I/σI	7.8 (2.0)	7.6 (1.6)
Completeness (%)	99.8 (99.9)	98.9 (99.6)
Redundancy	3.8 (3.7)	2.9 (2.9)
**Refinement**		
Resolution (Å)	1.76	2.50
No. reflections	67,730	22,873
*R*_work_/*R*_free_^3^ (%)	18.1/21.4	21.6/25.7
No. atoms		
Protein/RNA	3,695/906	3,600/755
Ligand / Ion / Water	13/6/648	27/2/105
B-factors (Å^2^)		
Protein	27.7	60.5
Ligand / Ion / Water	38.7/28.5/37.2	68.5/74.9/58.4
R.m.s. deviations		
Bond lengths (Å)	0.006	0.008
Bond angles (°)	0.83	1.05
Ramachandran stat.^4^	95.3/4.4/0.0/0.2	92.2/7.6/0.0/0.3

^1^One crystal was used for data collection for each structure.

^2^Values in parentheses are for highest-resolution shell.

^3^5% of data are taken for the *R*_free_ set.

^4^Values are in percentage and are for most favored, additionally allowed, generously allowed, and disallowed regions in Ramachandran plots, respectively.

### Structural identification of interactions between a fingers domain pocket and the 5′-end guanosine of the RNA template

**Figure 2.**
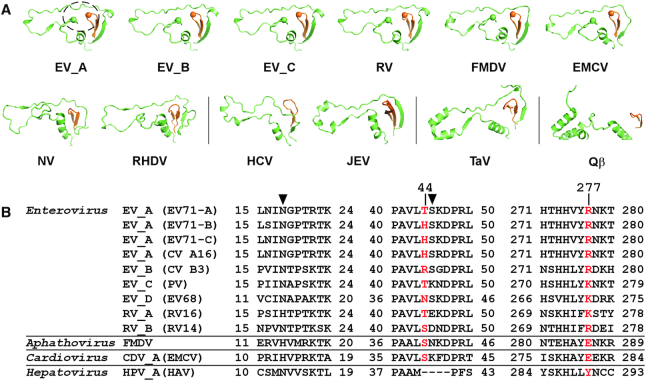
Structure and sequence conservation analyses of the nucleobase-binding pocket. Virus abbreviations: FMDV–foot-and-mouth disease virus; EMCV–encephalomyocarditis virus; NV–norovirus; RHDV–rabbit hemorrhagic fever virus; HCV–hepatitis C virus; JEV–Japanese encephalitis virus; TaV–Thosea asigna virus; Qβ–bacteriophage Qβ; HAV–hepatitis A virus. (**A**) Side by side comparison of the index/middle (in green/orange) fingers subdomains of RdRPs from different positive-strand RNA viruses derived from a superposition (least square methods) of the highly conserved RdRP motif C residues. The dashed circle in the EV_A structure indicates the location of the nucleobase-binding pocket. PDB entries used: EV_A–6KWQ, EV_B–3N6L, EV_C–1RA6, RV–1TP7, FMDV–1U09 and EMCV–4NYZ for *Picornaviridae* (top row); NV–1SH0 and RHDV–1KHV for *Caliciviridae*; HCV–1C2P and JEV–4K6M for *Flaviviridae*; TaV–4XHI for *Permutotetraviridae*; Qβ–3MMP for *Leviviridae*. (**B**) Sequence alignment of three regions constituting the pocket (first two are part of the index finger and last one is part of the middle finger) for RdRPs from the *Enterovirus* genus and three representative RdRPs from other genera of the *Picornaviridae*. Residues equivalent to the nucleobase stacking H44 and R277 in EV_A RdRP are shown in red, while those equivalent to the hydrogen bonding N18 and S45 are indicated by solid triangles.

Interestingly, the guanine moiety of the 5′-end guanosine of the downstream RNA was observed to interact with a fingers domain pocket, with its guanine plane sandwiched by the imidazole moiety of the index finger residue H44 and the guanidinium moiety of the middle finger residue R277 likely through stacking interactions (average distances to the guanine plane: 3.4 Å and 3.6 Å for guanidinium and imidazole moieties, respectively; angles between neighboring planes: 10–15°) (Figure 1B). Hydrogen bonding and other hydrophobic interactions involving index finger residues N18, T21 and S45 further stabilize the guanine base, with side chains of residue S45 interacting with the guanine-specific N2 position and residue N18 interacting with the purine-specific N7 position (Figure 1B, right). Although the crystal contacts between the downstream RNA and the neighboring ECs could contribute to the establishment and maintenance of these interactions, the delicate interaction network nevertheless indicates potential functional roles of this fingers domain pocket. This pocket comprises the tip of the middle finger, residues 18–21 and a unique ‘kink’ region (residues 44–55) of the index finger. Structurally, the kink region is highly conserved in RdRPs from the *Picornaviridae*, conserved to some extent in RdRPs from the *Caliciviridae*, but has not been found in RdRPs from other viral families in the positive-strand RNA viruses including the *Flaviviridae, Permutotetraviridae and Leviviridae* (Figure 2A). Sequence conservation analysis of representative picornavirus RdRPs indicates that residues corresponding to EV71 RdRP H44 and R277 are not well conserved, but the majority of residues at these two positions are able to provide hydrophobic interactions through their side chains (Figure 2B). We therefore hypothesized that this fingers domain pocket involving the structurally unique kink region may have been utilized by some picornaviruses to optimize their RdRP function through its capability of interacting with RNA bases.

### One-register in-crystal translocation does not disrupt the interactions between the 5′-end guanine and the fingers domain pocket

**Figure 3.**
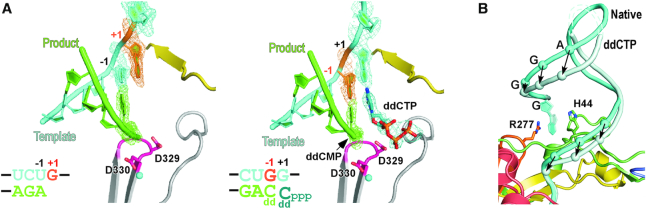
A comparison of the native and ddCTP-derived EC structures. (**A**) The EC structures are shown in cartoon representations with composite SA omit electron density map (contoured at 1.2 σ) overlaid for the native (left) and ddCTP-derived ECs. For clarity, the majority of the polymerase and RNA up- and downstream of the active site are not shown. Except for the templating +1 nt in the native complex and the −1 nt in the ddCTP complex are shown in orange to correlate the nucleic acids translocation, the coloring scheme is as in Figure 1. Magnesium ions are shown as cyan spheres. (**B**) A comparison of the downstream RNA of the two EC structures. The arrows indicate the movement of the backbone phosphates of equivalent nucleotides from the native (cyan) conformation to the ddCTP (light cyan) conformation. Dashed lines indicate the likely path of the disordered RNA in the ddCTP complex. RdRP structure is only shown for the native complex for clarity.

Some picornavirus RdRP EC crystal lattices allow in-crystal catalysis and translocation (10,43). As the first step to assess the interactions between the 5′-end guanine and the fingers domain pocket, we performed NTP soaking experiments to test whether progressive synthesis can ‘pull’ the 5′-end guanine out of the pocket. Based on the template sequence, CTP is the desired substrate in the next two NACs. However, the EC crystals soaked in CTP-containing solutions diffracted only poorly and did not yield useful structural data. With 2′,3′-dideoxy CTP (ddCTP) used in the soaking trials, we obtained a 2.5-Å resolution dataset (Table 1). The resulting structure was solved still in the C2 space group, albeit with apparent changes in the unit cell parameters. Compared with the original C2 lattice, the unit cell parameters a, c, and β are 5.7 Å (4.6%) longer, 5.9 Å (8.0%) shorter, and 3.4 Å wider, respectively. These observations indicate that the crystal lattice had adjusted itself to accommodate the ddCTP-induced polymerization reactions, while the CTP-soaking was likely too destructive to the lattice. In the ddCTP-derived structure, one ddCMP was incorporated into the product RNA chain and one translocation event had occurred, while the second ddCTP molecule was bound in the active site (Figure 3A, left). Consequently, the downstream RNA stem-loop underwent a small-scale twist as one nucleotide was pulled into the active site (Figure 3B and Supplementary Video S1). However, the 5′-end guanine was still buried in the pocket with most of the interactions maintained (Figure 3A, right). The incorporation of the ddCMP into the product chain prevented further catalysis and translocation due to the absence of a 3′-hydroxyl group, and therefore the disruption of the 5′-end guanine interactions with the fingers domain pocket may not be achieved through this approach. Nevertheless, the observation of the maintenance of these interactions and the twisting of the downstream RNA hairpin upon one translocation event together argue this type of interaction may be strong enough to serve as a regulatory factor of the polymerase.

### RdRP residue R277 is important to EV71 proliferation, while H44 may play an auxiliary role

**Figure 4.**
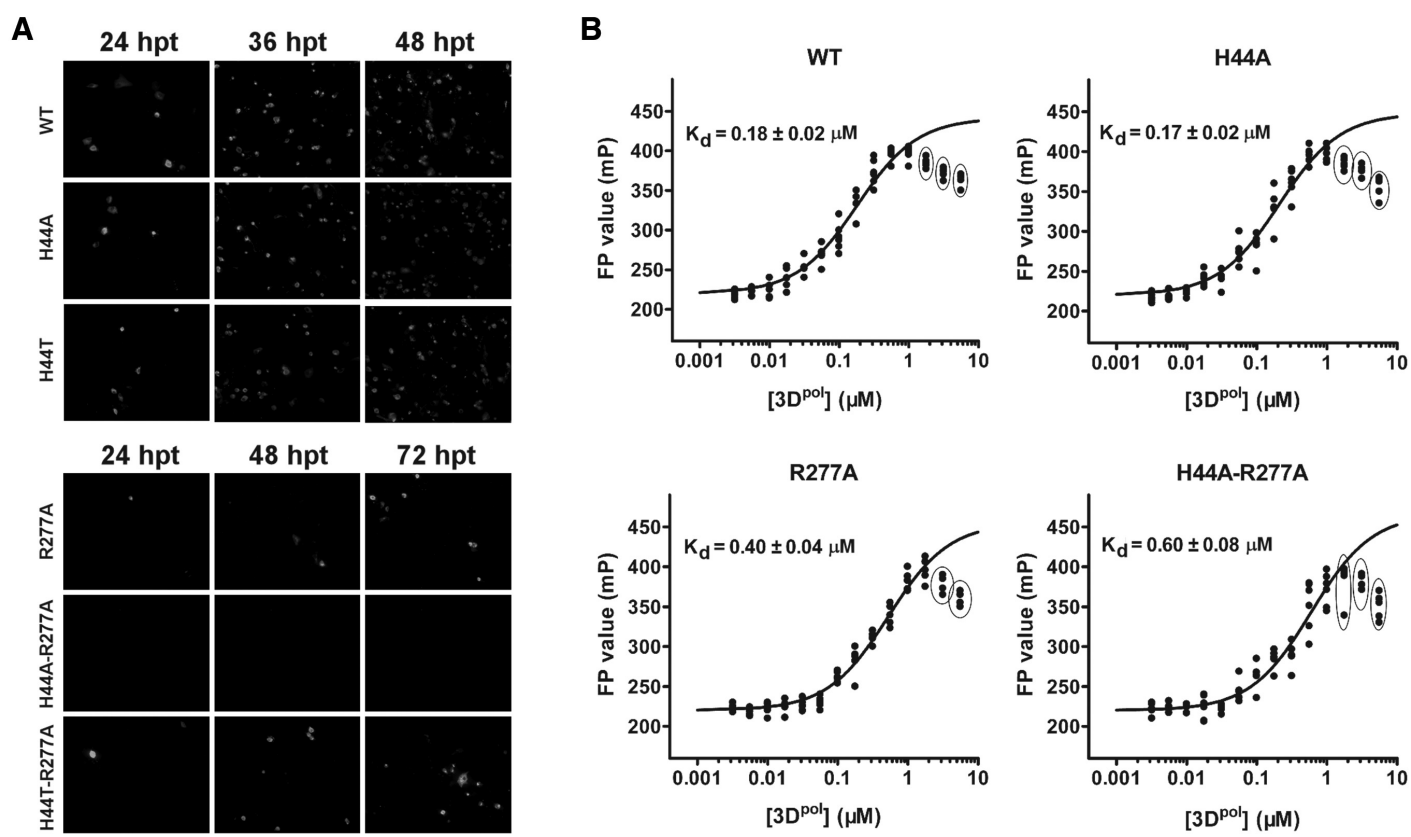
Mutations at the H44 and R277 sites affect virus proliferation and RdRP–RNA binding. (**A**) IFA analysis of genome-length EV71 viral RNA replication for WT and mutant viruses in transfected Vero cells at the indicated time points. (**B**) Binding of the T31/P8F RNA to the EV71_C 3D^pol^ constructs as detected by FP signal change. The dissociation constant (*K*_d_) derived from the data fitting following the one-site binding model was overlaid. Data points not included in the fitting routine were indicated by solid circles.

To assess the biological relevance of H44 and R277 in viral replication, we engineered a set of point mutations at these two sites into an infectious cDNA clone of EV71. Besides alanine mutations, a threonine mutation at residue 44 was also tested because threonine is the other naturally occurring amino acid at this position in EV71 (Figure 2B). After *in vitro* transcription, equal amount of the WT and mutant viral RNAs were transfected into Vero cells. Viral protein expression was compared between the WT and mutant viruses. An immunofluorescence assay (IFA) was used to monitor virus replication by detecting expression of the viral VP1 protein. As show in Figure 4A, all mutants except for the H44A-R277A showed IFA-positive signal at 24 h post-transfection (hpt), and the number of IFA-positive cells increased over time. There were comparable fractions of IFA-positive cells upon transfection with the RNAs of the WT, H44A and H44T (about 20, 50 and 80% positive cells at 24, 36, 48 hpt, respectively). In contrast, the R277A and H44T-R277A produced much fewer IFA-positive cells than the WT (about 1, 10 and 20% positive cells at 24, 36, 48 hpt, respectively), and no IFA-positive cells were observed at 72 hpt for H44A-R277A. These data collectively indicate that 3D^pol^ residue R277 is critical for the EV71 viral replication and viability, while residue H44 may play a synergistic role with R277.

### RdRP residue R277 showed an apparent effect on RNA binding ability, and H44 also contributed

To further dissect the molecular mechanism of how residues R277 and H44 contributed to virus proliferation, in particular related to the polymerase properties, we performed a series of biochemical characterizations comparing the WT and corresponding mutant RdRP proteins. RNA binding affinity was measured using an FP assay derived from a previous study (46). Various concentrations of WT or mutant RdRPs were incubated with the T31/P8 RNA construct bearing a fluorescein label at the 5′-end of the P8. Upon RdRP binding, the tumbling freedom of the fluorescent probe was reduced, resulting in an increase of the FP signal. The data were fitted to the one-site binding model to estimate the dissociation constant (*K*_d_). The results showed that WT, H44A, R277A and H44A-R277A RdRP had *K*_d_ values of 0.18, 0.17, 0.40 and 0.60 μM, respectively (Figure 4B). Alanine mutation of H44 alone does not weaken the RNA binding ability of RdRP, but could display a synergistic effect for the reduced binding ability of R277A. The variations in RNA binding affinities for WT and mutants are consistent with the data of virus proliferation. Note that the FP signals gradually decreased when polymerase concentrations were greater than 1 μM, possibly due to protein aggregation under relatively high concentrations (Figure 4B). We therefore omitted the data affected by this effect (circled dots in Figure 4B) in the fitting routine.

### R277A-containing mutants required longer time to reach equilibrium of forming binary complex with RNA

**Figure 5.**
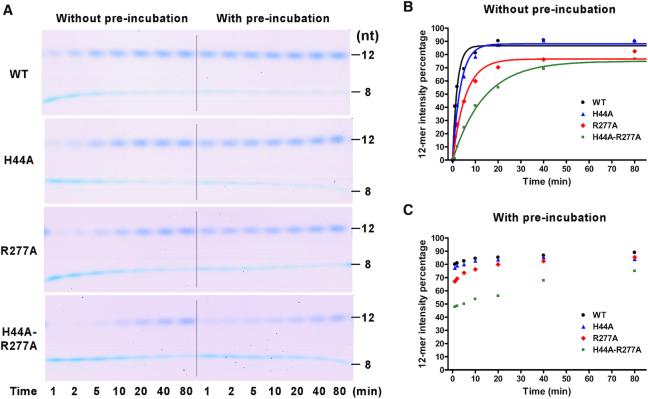
A comparison of EC formation of EV71_C RdRP constructs with or without protein–RNA pre-incubation. (**A**) Monitoring the 12-mer (P12) containing EC formation in a time course manner for four RdRP constructs with (left) or without (right) pre-incubation. (**B** and **C**) Quantitation analyses of EC formation with (B) or without (C) pre-incubation indicated by the 12-mer intensity percentage values (P12_int_/(P8_int_+P12_int_)). For each RdRP construct, the data in panel B were fitted to a single exponential rise model to aid the comparison.

The effects of RdRP residues H44 and R277 on EC formation were examined by monitoring the 12-mer production from T31/P8. In the presence of ATP and GTP, T31/P8 directed incorporation of a tetra-nucleotide GAGA to yield a 12-mer product. This is a multi-step process including RdRP–RNA binding and four rounds of nucleotide incorporation. If the process was initiated without a pre-incubation of RdRP and T31/P8, the 12-mer production exhibited a single-exponential behavior with the R277A and H44A-R277A mutants having slower production rates than the WT and H44A mutant (Figure 5A and B). We further conducted the RdRP and T31/P8 pre-incubation time test to determine the pre-incubation time required for each RdRP protein to reach an equilibrium while binding to the RNA construct, and applied these pre-incubation time periods in subsequent EC formation trials. After full pre-incubation, the 12-mer production was much faster than that without pre-incubation and the initial burst rates for all RdRP proteins were too fast to be detected in manual mixing experiments (Figure 5, compare panels B and C). These observations first indicate that the RdRP–RNA binding is the rate-limiting step in the conversion process. Secondly, the difference in the 12-mer production rate observed in the reactions without pre-incubation suggests that the R277A and H44A-R277A mutants likely have slower association rates with the RNA than the WT and H44A mutant have. The differences in the 12-mer amount after the initial burst of synthesis likely reflect different percentages of the RdRP–RNA binary complex formed after pre-incubation, and are consistent with the differences in *K*_d_ value determined in the FP assay (Figure 4B). Together with the FP data, these results suggest that the R277A-containing mutations affect both the RNA binding affinity and kinetics of the EV71 RdRP. We note that the usage of the Stains-All staining method in the quantitation analysis was shown as a valid approach at least for semi-quantitative analyses (21,31).

### Both H44 and R277 residues contributed to RdRP EC stability

**Figure 6.**
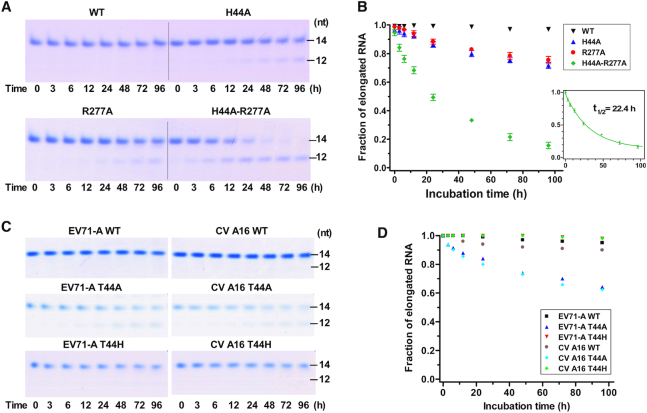
A comparison of EC stability of EV RdRP constructs. (**A**) Monitoring the 14-mer (P14) product formation by EV71-C RdRP ECs after a high-salt challenge for various time points. (**B**) Quantitation analysis of EC stability indicated by the intensity fraction values of the elongated RNA (P14_int_/(P12_int_+P14_int_)). Data obtained from the H44A-R277A construct were fitted to a single exponential decay model to estimate the EC half life (T_1/2_). Error bars indicate the SD values for measurements in triplicate. (**C**) Monitoring the P14 formation by EV71-A and CV A16 RdRP ECs after a high-salt challenge for various time points. (**D**) Quantitation analysis of EC stability same as in panel B for data presented in panel C.

Since the interactions between the 5′-end guanine and the fingers domain pocket were directly observed in the EV71 RdRP EC structure, an immediate question to ask is whether important properties of the EC are affected by these interactions. We first carried out a test of EC stability for WT and mutant RdRPs. The results showed that all ECs but the WT EC had gradually dissociated over time, as reflected by increasing fractions of non-extendable 12-mer products when treated with high-salt condition (300 mM NaCl) at 30°C over time (Figure 6A and B). WT RdRP formed the most stable EC, having nearly undetectable dissociation even after a 96-h treatment. The H44A and R277A mutants formed EC with moderately reduced stability, with about 28 and 25% EC dissociated after 96 h, respectively, while the H44A-R277A forms EC with the lowest stability, with 84% of EC dissociated at 96 h. By fitting the H44A-R277A data to a single exponential decay model, the dissociation rate constant (*k*_off_) of EC was estimated as 0.031 h^−1^, corresponding to an EC half life (t_1/2_) of about 22.4 h (Figure 6B). These data suggest that both H44 and R277 contributed to EC stability. Note that the stability of our EC is much higher than that of a PV RdRP EC obtained after incorporating two nucleotides (with a T_1/2_ of 3 h for WT PV RdRP EC) (38).

While the RdRP from the genotype-C EV71 (EV71-C) has a histidine at residue 44, those from genotype-A EV71 (EV71-A) and CV A16 have a threonine at this position (45). We introduced alanine or histidine mutation at residue 44 of RdRPs from these two viruses and assessed the EC stability along with corresponding WT proteins. The results showed that, for both RdRPs, both the WT and T44H could form highly stable ECs, while the EC derived from the T44A mutant showed moderately reduced stabilities (Figure 6C and D). Together with the virological data, these results suggest that both histidine and threonine at residue 44 could fulfill the function in stabilizing EC through the structurally observed interactions, with no significant differences between these two residue types.

### RdRP mutations at residues 44 and 277 do not apparently affect polymerase elongation rates and NTP substrate affinity

**Figure 7.**
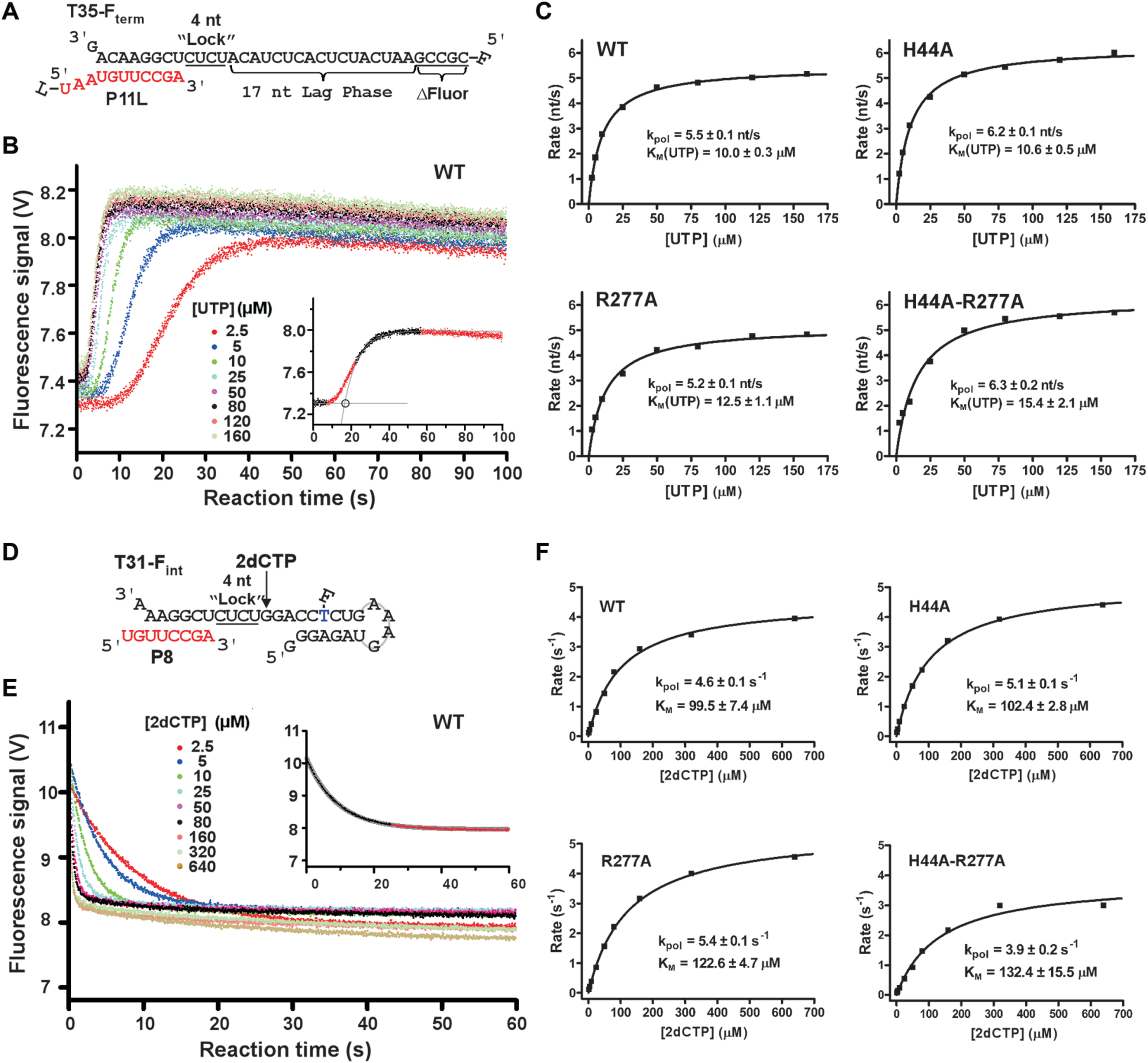
A comparison of elongation rate of EC formed by four represented EV71_C RdRP constructs. (**A**) The RNA constructs used to determine an average elongation rate constant. The 5′-fluorescein (F) labeled template (T35-F_term_) and the P11L primer were designed to have a 17-nt lag phase after a 4-nt incorporation (corresponding to the underlined CUCU ‘Lock’ template sequence) for EC assembly and before the fluorescence signal increase upon the last five nucleotides incorporation (ΔFluor). (**B**) Data from the WT construct showing a decrease in lag time with increasing concentration of UTP. Bottom right: a simple analysis was applied by fitting the latter part of the signal increase to a single exponential rise and by using the interception of this curve and the initial signal level to estimate the lag time for synthesizing the 17 nt. (**C**) The average elongation rates derived from data in panel B were fitted to the Michaelis–Menten equation to calculate the *k*_pol_ and *K*_M_ values for each construct. (**D**) The RNA constructs used to determine a single-nucleotide elongation rate constant. The internal fluorescein labeled template (T31-F_int_) and the P8 primer were designed to have an immediate fluorescence signal decrease upon a 2dCMP incorporation after a 4-nt incorporation (corresponding to the underlined CUCU ‘Lock’ template sequence) for EC assembly. (**E**) Data from the WT construct showing faster signal decrease with higher 2dCTP concentration. Top right: the data were fitted to a single exponential decay model to estimate the elongation rate with the latter part of the data omitted due to deviation of the single-exponential behavior. (**F**) The elongation rates derived from data in panel E were fitted to the Michaelis–Menten equation to generate the *k*_pol_ and *K*_M_ values for each construct.

We next assessed the elongation rate, another important property of a polymerase EC, for the WT and mutant RdRPs from EV71-C. Using fluorescence-based stopped-flow methods derived from previous kinetics studies characterizing enterovirus RdRPs (39,40), a T33F_term_/P11L (see ‘Materials and Methods’ section) RNA construct was used to measure the average elongation rates on a 17-nt stretch of the template (Figure 7A). The template T33F_term_ formed an eight base pair (bp) duplex with the P11L primer, and the EC was assembled after incorporating a GAGA tetra-nucleotide sequence by providing GTP and ATP as the only NTP substrates. The EC was then mixed in the stopped-flow instrument with all four NTPs, and the fluorescence signal only started to increase when the polymerase reached the fifth nucleotide to the 5′-end of the template (40) (Figure 7A and B). Therefore, the time of the fluorescence signal lag phase can be used to estimate the overall time for the polymerase EC to synthesize a total of 17 nt. By varying UTP concentrations in the NTP mixture, we were able to measure the average elongation rate constant (*k*_pol_) and the Michaelis constant (*K*_M_) for UTP (Figure 7B and C). The results showed that the WT and mutant RdRPs exhibited comparable *k*_pol_ (5.5–6.3 nt/s) and *K*_M_ (10.0–15.4 μM) values (Figure 7C). While these data suggest that the H44 and R277 may not affect the overall elongation rate in multiple rounds of nucleotide addition, we further estimated the enzyme parameters in a single nucleotide addition assay starting with a situation mimicking the EC formed by the T31/P8 in our structural study and ending with incorporation of a 2dCMP using a T31-F_int_/P8 construct (Figure 7D). By placing an internal fluorescein-labeled thymidine at the fifth position downstream of the incorporation site, the incorporation induces a decrease of fluorescence signal that can be fitted to a single exponential decay model. The results showed that the WT and mutant RdRPs also have comparable *k*_pol_ (3.9–5.4 s^−1^) and *K*_M_ (99.5–132.4 μM) values for single nucleotide addition (Figure 7E and F). While the *k*_pol_ values are comparable to those obtained in the multi-round addition reactions, the *K*_M_ values for 2dCTP is about 10-fold of those for UTP. Taken together, the stopped-flow kinetics data demonstrate that mutations at EV71 RdRP residues 44 and 277 do not significantly influence the elongation rate and NTP substrate affinity.

## DISCUSSION

The nucleobase-binding pocket identified in this study is formed between the ‘kink’ region of the RdRP index finger and the middle finger tip. In majority of the RdRP structures from the positive-strand RNA viruses, the index finger begins with a β-strand, arises to the top of the right-hand architecture, goes across the top to interact with the thumb tip and then folds back and descends to the starting point (Figure 2A). Although not directly participating in catalysis, the β-strand plays a structural role in forming a five-stranded β-sheet with the middle finger and the bottom part of the ring finger. The interactions between the tips of the index finger and thumb makes the encirclement of the right-hand architecture and may restrict large-scale movement of the fingers domain typically observed in other classes of single subunit polymerases for NTP repositioning toward catalysis (reviewed in (8)). Indeed, viral RdRPs utilize a small-scale rearrangement, mainly involving motifs A and D in the palm domain to close the active site (9–10,13). Collectively, the tip and bottom parts of the index finger play relatively conserved structural roles, while the middle part including the kink region may have the potential to vary in structure and function. Indeed, the kink region is only highly conserved in structure for the *Picornaviridae* RdRPs (Figure 2A, top row), is structurally varied in the *Caliciviridae* RdRPs (Figure 2A, bottom left), and is not present in RdRPs from the other three families with RdRP structures available (Figure 2A, bottom row, and excluding the *Caliciviridae*). Although structurally conserved, the residues responsible for nucleobase stacking and purine base recognition are only conserved in enteroviruses (stacking: T/H and R/K for EV_A residues 44 and 277 equivalents, respectively; recognition: N and S for residues 18 and 45 equivalents, respectively) (Figure 2B). Here we have shown that histidine and threonine can be exchanged by each other at EV_A residue 44 (Figure 6). In a previous study, a PV bearing a K276L mutation (equivalent to position 277 in EV71_A RdRP) in the RdRP region was found to revert to WT phenotype with an arginine replacing the leucine (47), suggesting that arginine and lysine are interchangeable at this position. Interestingly, when the same site was mutated to alanine (K276A) in another study, the mutant PV was found to produce RNAs with shorter poly(A) tails (48). With the role of EC stability contribution considered, it is conceivable that an alanine mutation at this position could alter the reiterative polyadenylation process. In order to assess the contribution of the hydrogen bonding interactions between the pocket and the terminal nucleotide, we mutated S45 to a phenylalanine (S45F) or a leucine (S45L) and found that the EC stability was also impaired with these mutations, albeit to different extents (Supplementary Figure S1A). It is also worth noting that the intra-complex interaction between a protein surface pocket and a base within an RNA tightly bound to that protein may be advantageous if compared to interactions between the same pocket and a base-containing small molecule. When a GG dinucleotide was provided at 10 or 100 μM (2.5- or 25-fold of the T31 RNA concentration) in the EC stability assay as a challenging agent, no obvious inhibitory effect was observed (Supplementary Figure S1B).The regulatory function of the nucleobase-binding pocket identified in this study may only be utilized by a small group of picornaviruses. However, similar regulation mode through surface pocket-nucleic acid interactions may occur in nucleic acid motor enzymes as suggested below. Note that W5, a residue within the index finger β-strand of PV RdRP, was shown to modulate EC stability and was proposed to interact with the RNA template through stacking interactions (38). Structurally, PV RdRP W5 interacts with a metal ion mediated residue cluster comprising H270, H272 and C281 in the middle finger β-strands (9). Though a similar nucleobase-binding pocket was not structurally observed, W5 and its neighboring residues could offer hydrophobic interactions necessary for nucleobase binding. Although lacking direct structural evidence, it is possible that W5 may achieve its contribution in EC stability through an interaction mode analogous to what is observed in the current study. While both the W5 and nucleobase-binding pocket are located at the downstream side of the RNA, a previously characterized basic patch within the RNA template entry channel is at the upstream side interacting with the template backbone phosphates (49), not adjacent to neither downstream site mentioned above (Supplementary Figure S2).

**Figure 8.**
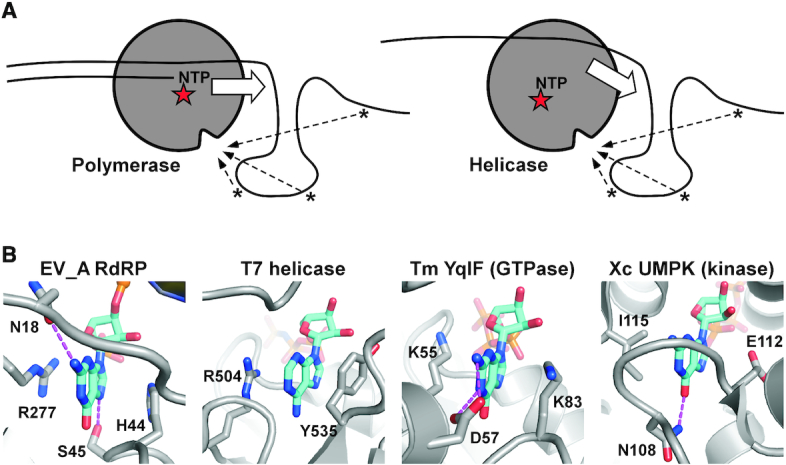
Similar nucleobase-binding pocket may be generally present in nucleic acid motor enzymes. (**A**) Working models showing interaction opportunities (dashed arrows) between the nucleobases in the nucleic acids and the nucleobase-binding surface pocket in nucleic acid motors such as polymerases (left) and helicases (right). The red stars indicate the enzyme active sites. Block arrows indicate the moving direction of the enzymes. (**B**) A comparison of the nucleobase-binding pocket identified in this study and those observed in other nucleobase-binding proteins (not limited to nucleic acids motor enzymes) with similar sandwich-like stacking interactions. The nucleotides and the side chains of protein residues participating in nucleobase stacking and hydrogen bonding (dashed lines) interactions are shown in sticks. PDB entries used: EV_A RdRP–6KWQ; T7 helicase–1E0J; Tm (*Thermotoga maritima*) YqlF–3CNN; Xc UMPK (*Xanthomonas campestris* uridylate kinase)–3EK5).

NTP-driven nucleic acid motor enzymes, such as polymerase, helicase and translocases, utilize the energy provided by phosphoryl transfer or NTP hydrolysis reactions to move along nucleic acids. Typically in these proteins, the NTP binding pocket is the active site while the surface of the protein is accessible to the interactions with the nucleic acids up- or down-stream of the active site. Although not always essential to the enzymatic activities, the sequence-non-specific interactions between these proteins and the nucleic acids beyond the active site could contribute to the processivity of these enzymes, because they provide extra anchoring point(s) to the nucleic acids (Figure 8A). In this work, we identified, in EV_A RdRPs, unique interactions of this category with a small pocket suitable for nucleobase binding that captured the downstream RNA and contributed to the stability of the RdRP EC. Introducing mutations at two residues responsible for the stacking interactions with the nucleobase resulted in reduced levels of virus proliferation, suggesting that this type of interactions likely occurred in the viral genome replication process. During viral genome replication, a replicating RdRP could encounter a variety of RNA structural forms (e.g. double stranded, single stranded, etc.) with or without nucleobases accessible to such a binding pocket. Therefore, this type of interaction may only occur occasionally or randomly during the genome replication process. However, once occurring, it probably improves the stability of the RdRP–RNA complex and therefore makes positive contribution of the processive synthesis of genome-length RNA. When the unpaired 5′-guanosine was not present, as in the EC formed using a construct with a 30-mer template (T30), the EC stability was only slightly reduced comparing to that formed using the T31/P8 construct for the WT RdRP (Supplementary Figure S3). When the H44A-R277A mutant was combined with the T30/P8 construct, the EC stability was also apparently affected (Supplementary Figure S3). These data suggest that either a breathing nucleotide (i.e. being in an equilibrium between the paired and unpaired states) or other unpaired nucleotide can bind to the fingers domain pocket and contribute to EC stability. We further proposed that similar interactions may commonly occur in NTP-driven nucleic acid motors. The fact that similar interactions were not previously identified may be related to the randomness of the interaction sites along the nucleic acids. The inter-EC interactions in our crystal lattice that stabilize the downstream RNA played a key role for identifying this type of interactions, inspiring us to collect further evidence supporting its role in enzyme stability. In the DNA-dependent T7 RNA polymerase EC, residues E168, R647 and N671 form a platform and allow the +1 non-template nucleotide to bind, possibly contributing to the maintenance of the downstream edge of the transcription bubble (50) (Supplementary Figure S4A). If compared to the T7 RNA polymerase EC, the 5′ nucleotide in our EV71 RdRP EC is equivalent to the +3 non-template nucleotide, and therefore these two interaction modes are analogous but not identical (Supplementary Figure S4B). The sandwich-like nucleobase binding interactions observed in our study are very common in nucleotide binding enzymes (not to be confused with the aforementioned NTP-driven nucleic acid motors) including helicases (51), GTPases (52) and kinases (53), while the residue types providing the hydrophobic interactions to the nucleobase are quite diverse (Figure 8B). Hence, it is not difficult for nucleic acid motors to assemble such a nucleobase-binding pocket during evolution. Once functional contribution, such as processivity or stability enhancement, is established based on the interactions between the pocket and nucleobases, key residues constituting the pocket could become more conservative as positions 44 and 277 in enterovirus RdRPs.

Members of the *Enterovirus* genus include causative agents of HFMD, poliomyelitis and common cold. The identification of the functionally conserved nucleobase-binding pocket in this study may provide opportunities for developing inhibitors or live attenuated vaccines. As discussed above, the assembly of this surface pocket is analogous to nucleotide binding pocket observed in other enzymes. Therefore, it may have the potential to bind not only nucleobases within a nucleic acid strand, but also nucleotide analogs commonly studied in antiviral research for RNA viruses. With respect to the development of live-attenuated vaccine, this pocket has two advantages. First, the two key residues are far apart in the primary structure, and therefore it is less susceptible to reversion if both sites are chosen for mutation in the vaccine strain. Second, the residue type for both positions is not strictly conserved as long as it can provide suitable hydrophobic interactions to the nucleobase, and allows a variety of amino acid combinations to be tested for viable viruses with attenuated features.

In summary, we identified a nucleobase-binding pocket in enterovirus RdRP by crystallography and analyzed its relevance to polymerase function through *in vitro* biochemical characterization and cell-based virological studies. Taken together, this pocket contributes to RdRP EC stability and virus proliferation, likely through its nucleobase binding capability. We propose that similar regulatory mechanisms, although rarely reported, may commonly exist in nucleic acid motor enzymes. Our work could serve as a general reference for future studies to dissect the mechanism behind nucleobase anchoring sites identified in these enzymes through non-crystallographic approaches.

## DATA AVAILABILITY

Atomic coordinates and structure factors for the reported crystal structures have been deposited with the Protein Data bank under accession numbers 6KWQ and 6KWR.

## Supplementary Material

gkz1170_Supplemental_Files
