# Supplementary material for: A nucleobase-binding pocket in a viral RNA-dependent RNA polymerase contributes to elongation complex stability
Source: Nucleic Acids Res. 2019 Dec 21;48(3):1392–405. doi: 10.1093/nar/gkz1170 (PMC7026628; doi:10.1093/nar/gkz1170)
Supplement: gkz1170_Supplemental_Files [file gkz1170_supplemental_files.zip › supplementary_data_updated.pdf]

Supplementary Data for

“A nucleobase-binding pocket in a viral RNA-dependent RNA polymerase contributes to elongation complex stability”

by

Wei Shi[1,2,#], Han-Qing Ye[1,#], Cheng-Lin Deng[1], Rui Li[1,2], Bo Zhang[1,], Peng Gong[1,3,*]

1 Key Laboratory of Special Pathogens and Biosafety, Wuhan Institute of Virology, Center for Biosafety Mega-Science, Chinese Academy of Sciences, No.44 Xiao Hong Shan, Wuhan, Hubei, 430071, China

2 University of Chinese Academy of Sciences, Beijing, 100049, China

3 Drug Discovery Center for Infectious Diseases, Nankai University, Tianjin 300350, China

# These authors wish it to be known that, to their opinions, the first two-authors should be regarded as Joint First Authors.

* To whom correspondence should be addressed: Tel/Fax: +86 27 87197578; Email: gongpeng@wh.iov.cn.

Supplementary Figures and Figure Legends

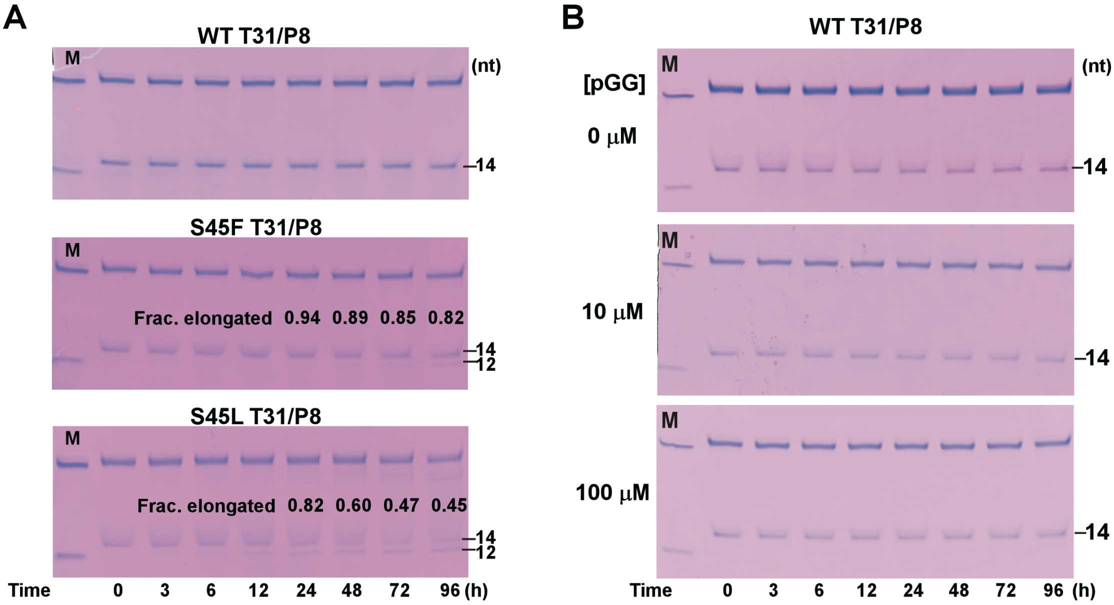

Figure S1. EV71-C RdRP elongation complex (EC) stability tests with two S45 mutants and a challenging GG dinucleotide. A) Mutations at the RdRP S45 site affect EC stability. Two different mutants, S45F and S45L supposedly causing steric clash upon nucleobase binding to the fingers domain pocket, were compared with WT RdRP in the EC stability assay using the standard T31/P8 RNA. An EC with a 12-mer product (EC12) produced in a standard EC formation reaction was used as a marker (depicted with "M"). The intensity fraction values of the elongated RNA ($P14_{int}/[P12_{int}+P14_{int}]$) were quantified and the critical data were presented. B) A GG dinucleotide with a 5′-monophophate (pGG) has little effect on EC stability. Three different concentrations (0/10/100 µM) of pGG were tested and the EC was formed using WT RdRP and the T31/P8 RNA.

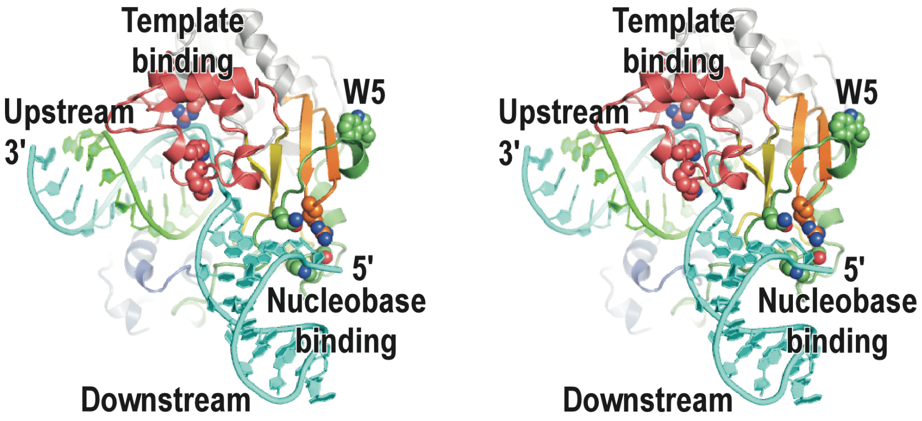

Figure S2. The spatial relationship between the nucleobase-binding pocket and previously identified RdRP elements involved in RNA binding. Stereo-pair images of the EV71-C RdRP EC with key side chains of the nucleobase-binding pocket, a basic patch participate in template binding, and residue W5 shown in spheres. The coloring scheme is the same as in Fig. 1A.

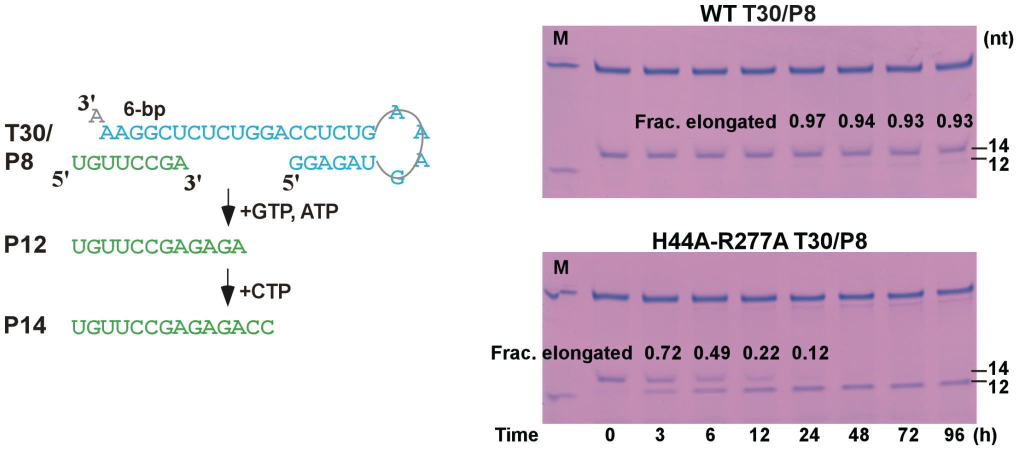

Figure S3. EV71-C RdRP EC stability test with a 5′-truncated RNA template T30. EC formed by the T30/P8 construct may be capable of binding to the fingers domain pocket. Left: A diagram of the T30/P8 construct and the reaction scheme. Right: Monitoring the 14-mer (P14) product formation by EV71-C RdRP ECs using the T30 template after a high-salt challenge for various time points. The intensity fraction values of the elongated RNA ($P14_{int}$/[$P12_{int}$+$P14_{int}$]) were quantified and the critical data were presented.

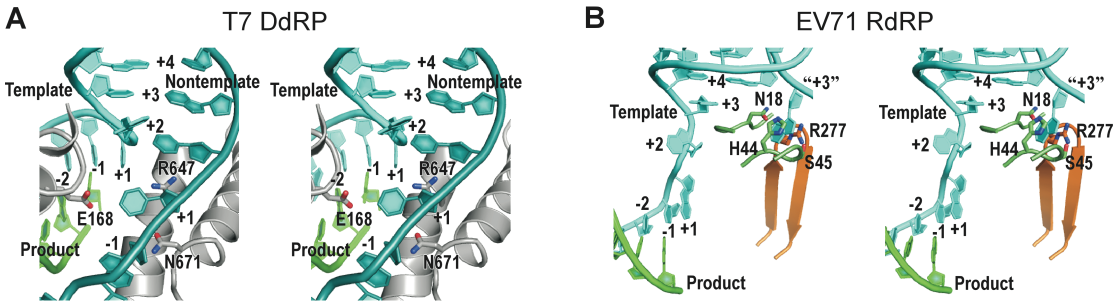

Figure S4. A comparison of the nucleotide binding element in T7 RNA polymerase and the nucleobase-binding pocket in EV71-C RdRP. A) Stereo-pair images of the DNA-dependent T7 RNA polymerase (T7 DdRP) highlighting the interaction between the +1 nontemplate nucleotide and three residues (E168, R647, and N671). Coloring scheme: T7 RdRP in grey, template in cyan, product in green, and nontemplate in deep teal. B) Stereo-pair images of the EV71-C RdRP highlighting the interaction between the 5′-nucleotide (corresponding to the +3 nontemplate nucleotide in T7 DdRP). The coloring scheme is the same as in Fig. 1A. For both panels, key side chains are shown in sticks. PDB entries: T7 DdRP–1S76; EV71 RdRP–6KWQ.

Supplementary Movie Legends

Movie S1. A modeled conformational transition of the downstream RNA upon 2′,3′-dideoxy CTP (ddCTP) incorporation. The movie starts with the EV71-C RdRP EC native structure (PDB entry 6KWQ), finishes with the ddCTP-derived EC structure (PDB entry 6KWR). The RdRP chains in the structures were superposed using the least-square methods. The coloring scheme is the same as in Fig. 1A. The α-carbon atoms of residues H44 and R277 were shown as large spheres to highlight the location of the nucleobase-binding pocket.
